# Temporal Synergies Detection in Gait Cyclograms Using Wearable Technology

**DOI:** 10.3390/s22072728

**Published:** 2022-04-02

**Authors:** Marija M. Gavrilović, Milica M. Janković

**Affiliations:** School of Electrical Engineering, University of Belgrade, Bulevar kralja Aleksandra 73, 11000 Belgrade, Serbia; piperski@etf.rs

**Keywords:** gait, gait cycle, ground reaction force, inertial measurement unit, principal component analysis, stroke, synergy, wearable device

## Abstract

The human gait can be described as the synergistic activity of all individual components of the sensory–motor system. The central nervous system (CNS) develops synergies to execute endpoint motion by coordinating muscle activity to reflect the global goals of the endpoint trajectory. This paper proposes a new method for assessing temporal dynamic synergies. Principal component analysis (PCA) has been applied on the signals acquired by wearable sensors (inertial measurement units, IMU and ground reaction force sensors, GRF mounted on feet) to detect temporal synergies in the space of two-dimensional PCA cyclograms. The temporal synergy results for different gait speeds in healthy subjects and stroke patients before and after the therapy were compared. The hypothesis of invariant temporal synergies at different gait velocities was statistically confirmed, without the need to record and analyze muscle activity. A significant difference in temporal synergies was noticed in hemiplegic gait compared to healthy gait. Finally, the proposed PCA-based cyclogram method provided the therapy follow-up information about paretic leg gait in stroke patients that was not available by observing conventional parameters, such as temporal and symmetry gait measures.

## 1. Introduction

The central nervous system (CNS) controls many degrees of freedom (DOFs) of the musculoskeletal system, coordinating many muscle activities on many joints. Human movements can have different trajectories, speeds, and accelerations even when they achieve the same goal. To control so many DOFs, it becomes necessary for the CNS to have a complex and delicate organizational structure [1]. Different mathematical approaches for modeling realistic multi-joint movements were suggested in the literature, based on the various optimization functions such as minimum jerk [2,3], minimum torque change [4], minimum effort [5], as well as more complex functions [6,7]. An organizational approach based on activities of functional groups (called synergies) was also suggested [8]. Synergies represent patterns of body segment coactivations. Researchers have hypothesized that the nervous system activates synergies by a neural signal and creates a set of temporal–spatial synergy modules. These modules represent a smaller dimensional space than the space formed by individual DOFs. Synergies can be found at various levels, such as joint coordinates or muscles [9,10]. Kinematic synergies may result from muscle synergies, i.e., as a consequence of muscle activity [11,12]. In addition, researchers have suggested that CNS develops synergies to execute endpoint motion [13,14]. Motor intra-limb coordination is the ability to coordinate segments in a sequence [15]. This coordination can be accomplished by controlling the endpoint trajectory.

The human gait can be described as a synergistic activity of all individual components of the human sensory–motor system. Different mathematical models of muscle synergies are known in the literature: invariant temporal (“temporal synergies”), spatial (“synchronous synergies”), and spatiotemporal (“time-varying synergies”) [16]. All of these approaches reduce the dimensionality of the movements, but they are not equivalent to each other.

Temporal muscle synergies imply the existence of a set of temporal components common to different activation tasks [17,18]. Ivanenko et al. [19] showed that there is a basic set of five temporal components extracted from recorded electromyography (EMG) signals in controls and patients with a spinal cord injury (SCI). The consistent timing of motor patterns across various walking tasks was shown even with considerable variation of muscle coactivation. These temporal components represent the timings of the intersegmental coordination and may reflect a neural strategy for coordination in a low dimensional set of patterns that facilitate control of gait. Furthermore, these timings are argued to represent a control variable in central pattern generators [20]. Furthermore, it was reported that the nervous system’s activation pattern during walking does not depend on walking speed, including running [21].

However, EMG analysis has certain limitations; for example, adipose tissue can affect EMG recordings. There is also the problem of muscle crosstalk and a lack of deep muscles reliability [22]. Furthermore, it was shown that the synergy structure is dependent on the number and choice of muscles [23]. On the other hand, the intermediate dynamic representation is a logical connection between highly variable muscle activity and whole-body mechanics [20].

The synergism in people without sensorimotor impairment differs from patients with sensorimotor disorders. Injury to the CNS, such as stroke, leads to changes in gait modality and synergism [24]. These differences can be observed concerning the parameters that characterize gait. Characteristics of gait in stroke hemiplegic patients are: decreased speed, decreased and asymmetrical step length, decreased stance and single support times on the affected side, changes in joint kinematics, and overall asymmetry in different metrics [25]. The rehabilitation process restores the gait, i.e., retrains the patient to stand and walk with reduced sensory–motor resources and to walk in the way most similar to the gait before the disorder. During rehabilitation, it is essential to objectively quantify the success of the applied protocols and therapies on gait performance.

The gold standard for quantitative gait analysis implies the usage of high cost, space, and time-consuming 3D motion capture systems and force platforms [26,27]. Recently, the development of wearable technology enabled the usage of alternative low-cost approaches for gait assessment based on inertial measurement units (IMU) and ground reaction force (GRF) sensors [28,29]. These portable, wireless systems are suitable for clinical and home monitoring [30]. They are easy to use, non-invasive, small, compact, and robust enough to provide valuable information for the objective evaluation of the gait performance of people with neurological disorders [31,32]. Conventionally, the prerequisite for quantitative gait analysis is gait segmentation. Several algorithms were developed to tackle this problem in IMU-based systems, such as zero-crossing and threshold methods [33,34]. However, these algorithms usually have lower accuracy in pathological gait [35]. The gold standard for gait phase partitioning is the measurement signal of the direct contact between the foot and the ground. For this reason, some wearable systems, in addition to the IMUs, also contain foot pressure insoles in shoes. However, gait phases’ detection accuracy and reliability also depend on the location of the GRF sensors [36]. It is difficult to determine the heel-strike events automatically in the recordings of the person after a stroke, precisely because of the problem with the drop foot [37]. Thus, a gait analysis methodology that does not need the segmentation process is preferred.

The principal component analysis (PCA) has been widely used to discover “hidden” patterns in the high dimensional space of human gait signals in healthy and pathological gait [19,38,39]. Many researchers used PCA to identify muscle [21] and limb synergies [40], which are proposed to be building blocks for motor behavior [19]. Recently, it was shown that the space of two-dimensional PCA cyclograms allows simple assessment of gait performance in stroke hemiplegic patients [41].

This study aims to evaluate whether invariant (temporal) features of synergies can be extracted by analyzing foot (endpoint) dynamics (kinetics and kinematics) acquired by a wearable device, without the need for gait segmentation and need of EMG data acquisition. PCA was applied on the signals acquired by wearable sensors (IMU and GRF integrated into shoe insoles and mounted on feet) to detect temporal synergies in the space of two-dimensional PCA cyclograms. The idea of invariant temporal components “hidden” in motion dynamics signals was explored, as shown in the literature for EMG signals [19]. To test the hypothesis about invariant temporal dynamic synergies, the gait was analyzed at different speeds in healthy subjects. The gait of stroke hemiplegic patients before and after the rehabilitation therapy was also analyzed. The differences between healthy and pathological gait patterns were observed concerning the parameters which define the temporal dynamic synergies. Additionaly, it was investigated whether the method for detecting temporal dynamic synergies from IMU and GRF signals has an additional practical value for the paretic side recovery follow-up of stroke patients compared to the conventional gait analysis results, such as symmetry and temporal gait parameters.

## 2. Materials and Methods

### 2.1. Subjects

Nineteen subjects took part in this study: 14 healthy persons (without sensory–motor deficiency) and five hemiplegic stroke patients in the subacute phase (4–6 months after stroke). Subject characteristics are shown in Table 1. The patients could follow instructions from clinicians. The patients could walk with or without cane support.

The patients participated in functional electrical stimulation (FES)-based therapy. The effectiveness of FES therapy for the drop foot correction was assessed by observing the neuroplasticity changes using electroencephalography examination. Eight-channel MOTIMOVE electronic stimulator (3F—FIT FABRICANDO FABER, Belgrade, Serbia, [42]) was used for FES therapy, augmenting the patient’s pedaling (OMEGO^®^ Plus, Graz, Austria, [43]). The duration of the rehabilitation protocol was four weeks. The healthy subjects did not participate in the FES therapy.

The experimental design was approved by the ethical review board of the Rehabilitation Clinic “Dr Miroslav Zotović” in Belgrade. Participants were well-informed about the noninvasive protocol and they signed informed consent forms prior to gait assessment.

### 2.2. Instrumentation

**Figure 1:**
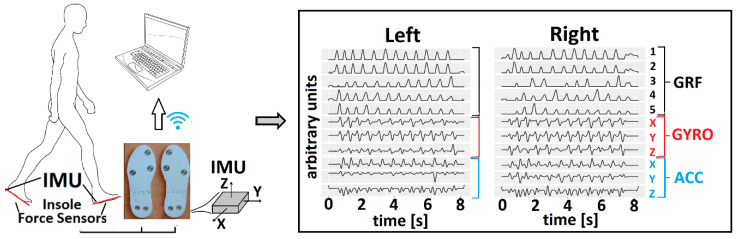
Gait Teacher instrumentation and output signals from both insoles.

The Gait Teacher (RehabShop, Belgrade, Serbia) [44] was used in the study. This system comprises 10 GRF sensors (five per foot insole) that measure vertical forces and two IMUs (MPU6050 module) with integrated three-axis accelerometers and gyroscopes into the insoles. Each foot insole has two piezoresistive GRF sensors in the heel zone (medial heel—HeelM, lateral heel—HeelL), two sensors in the metatarsal (medial metatarsal—MetaM, lateral metatarsal—MetaL), and one sensor in the toes zone (Toe). Each sensor can estimate pressure up to 3.5 MPa. The characteristics of GRF sensors are: linearity < ±0.25% FS, BFSL, repeatability < ±0.075% FS, hysteresis < ±0.05% FS, zero thermal error < 0.75% FS, @35 °C, span thermal error < 0.75% FS, @35 °C, and stability error < ±0.2% FS/year. The gyroscope and accelerometer specifications within IMU are: supply voltage 2.3–3.4 V, consumption 3.9 mA, calibration tolerance ±3%, I2C interface support, and operating temperature −40 °C to −85 °C. The IMU can measure the 3D acceleration (range of ±4 g) and the 3D angular velocity (range of ±500 deg/s). The 3D directions in the IMU are as follows: the *z*-axis directs up from the insole, the *x*-axis directs ahead, and the *y*-axis directs medially. The direction of angular velocity ω_x_ is from heel to toe. This angular rate is perpendicular to the insole (frontal plane). The rate ω_y_ is also orthogonal to the insole and directed laterally. The angular rate ω_z_ is in the plane of the insole pointing up. Each insole is wirelessly connected to the computer. Eleven signals from each insole are transferred at a sampling rate of 100 Hz. The acquisition software was built in LabView (National Instruments, Austin, TX, USA). The built-in software synchronizes IMU and GRF sensors. The system provides data with a time delay of 20 ms. Data are stored in text format (.txt) for further offline analysis (Figure 1). In conclusion, the data obtained with the Gait Teacher are a set of five GRF time series and six-time series of angular velocities and accelerations per insole. The output is a large matrix with 22 components [41].

### 2.3. Experiment Protocol

The Gait Teacher insoles were fitted to the subjects’ shoes. First, the outputs from GRF sensors were zeroed: a participant raised the left foot and then the right and held it in the air for about 2 s (no load) while the clinician pressed the set button on the host computer. The IMU signals were zeroed while the participant stood on both feet for about 2 s.

**Figure 2:**
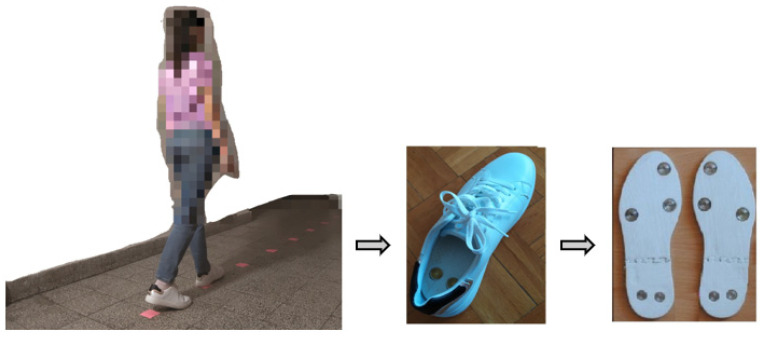
An example of the experimental setup.

Healthy subjects walked on a flat surface 10 m long. Before the recording, the respondent practiced walking for a few minutes. Signals from all sensors were recorded from three consecutive sessions. They walked at different speeds: 0.4 m/s, 0.8 m/s, 1 m/s, 1.6 m/s, and 2 m/s. The lowest speed was chosen to mimic the speed of the patients after stroke in the subacute phase ~0.4 m/s [45]. The highest speed was set to be the highest speed of the oldest participant. The oldest participant in the study was a healthy individual, 70 years old, and the maximal speed for this age is ~2 m/s [46]. Different speeds were recorded to address the diversity of different gaits and therefore generalize results from temporal synergies detection as much as possible, controlling for speed. To ensure a particular gait speed on the ground (avoiding the treadmill effect on the gait performance [47]), the subject followed the sound of the metronome, which signaled the cadence depending on the desired walking speed (Table 2). Markers were placed at one of the predefined distances: 0.5, 0.75, and 1 m, Figure 2. The markers were not moved between consecutive gait sessions of one participant for the same walking speed. This distance between markers was changed depending on the height or walking speed of the participant, so that the subject feels comfortable while walking. For higher subjects or higher speeds, markers were set at a greater distance. The metronome signaled the beginning of each stride, which occurred at specific markers on the floor. Table 2 shows the cadences required for different speeds, on a path of 10 m, for three possible stride lengths (the most suitable one for a particular respondent, heuristically chosen depending on the subject’s height and the specified speed).

The patients were asked to walk at a self-selected preferred speed. Signals from all sensors were recorded from three consecutive sessions. The rest between runs was about 1 min long. The clinician could monitor the signals on the computer screen during the recording. Signals recorded from sensors mounted on the paretic leg before therapy (p.b.), nonparetic leg before therapy (np.b.), paretic leg after therapy (p.a.), and nonparetic leg after therapy (np.a.) were separately analyzed. The number of strides performed by healthy subjects was 534, 534, 400, 366, and 300, respectively for speeds: 0.4 m/s, 0.8 m/s, 1 m/s, 1.6 m/s, and 2 m/s. The number of strides performed by patients was 110 before and 168 after therapy.

### 2.4. Data Preprocessing

**Figure 3:**
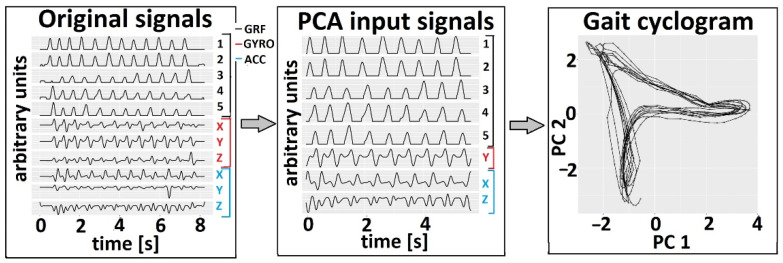
PCA cyclogram generation in the first two principal components space. Example data are from a person with no known sensory–motor impairment. The original signals were acquired by the Gait Teacher system (**left panel**). The subset of signals from the whole gait session (**middle panel**) was used to form a cyclogram (*PC*_2_ vs. *PC*_1_, **right panel**).

The first and last strides were excluded from the gait analysis since the person needs to adapt the gait speed to the sound of a metronome. The signals were filtered by a low-pass Butterworth filter, third order, with a cut-off frequency of 5 Hz [48]. Signals obtained by sensors from different legs were analyzed separately. The input for PCA included five signals from five GRF sensors, angular velocity in the sagittal plane, Gyro_Y, and accelerations in the frontal plane, Acc_X, and transverse plane, Acc_Z, (in total, eight signals per leg, Figure 3). They are chosen heuristically because the gait is predominant in the profile plane. All PCA and statistical analyses were done in the R software environment, version 3.5.1.

### 2.5. Detection of Temporal Synergies

PCA was used to find common temporal components hidden in the waveforms of dynamics’ signals. The PCA input signals were normalized to have unit variance. Bartlett’s sphericity test showed that the signals were suitable for PCA [49]. The PCA allowed the mapping of original data into the orthogonal space, where the principal axis is the direction of the data’s maximal variance [50].

The analysis included calculating the correlation matrix, extracting the principal component of the varimax rotation, and calculating factor scores. These factor scores can be interpreted geometrically as the projections of the observations onto the principal components [49]. The whole preprocessed gait session (gait cyclogram) per subject was input for PCA. Therefore, the standardization across subjects with a different range of motions (subjects may engage in different walking strategies) was avoided [41]. After PCA, no stride segmentation was performed. Consequently, there was no need for the time interpolation of the signals for separate gait cycles.

The proposed method uses 2D gait cyclograms to represent recorded foot dynamics in the space of the first two principal components, *PC*_1_ and *PC*_2_ (Figure 3). The repetitive nature of near-cyclic events resulted in the overlapped cyclogram (cyclograms of gait cycles were overlapped) [41]. The calculation of principal components’ quantitative parameter of cyclogram, introduced in [41], is shown in Equation (1) and expressed as an angle *θ* in each time point (observation).

(1) $$\theta =arctg\frac{PC_{2}}{PC_{1}},$$

where *PC*_1_ and *PC*_2_ are the coordinates of the observations on the first two principal components (*PC*).

**Figure 4:**
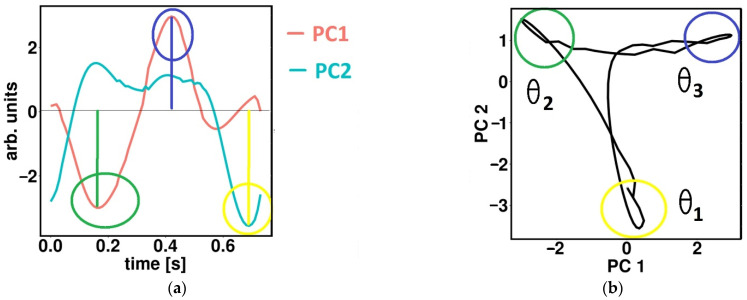
Representation of temporal synergies on the first two components on: (**a**) time PC signals; (**b**) PCA gait cyclograms. Time activations of temporal synergies are displayed using different colors (green, blue, yellow).

Figure 4a shows examples of specific time points where three temporal components exist during the single gait cycle by different colors (green, blue, yellow). These points correspond to the local extremums of *PC*_1_ or *PC*_2_. In Figure 4b the corresponding points of temporal synergy are presented in gait cyclogram using the same colors as in Figure 4a. The corresponding angles *θ* of observations that belong to each of three temporal components are marked by *θ*_1_, *θ*_2_, and *θ*_3_.

**Figure 5:**
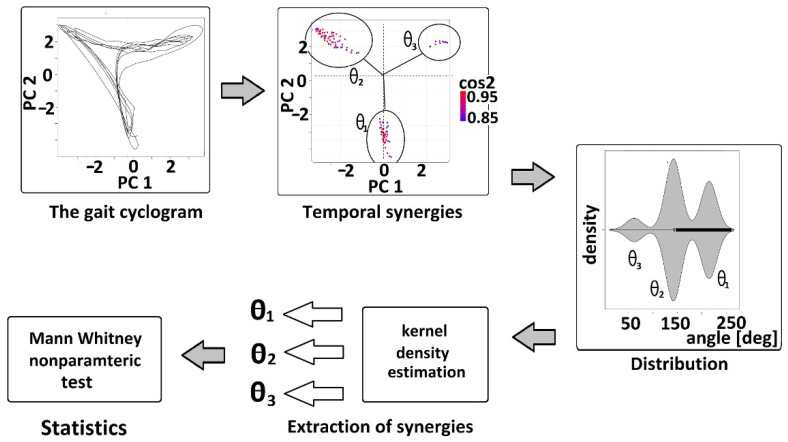
Temporal synergies detection from the PCA gait cyclograms.

The overall schema of the performed methodology on the PCA gait cyclogram is shown in Figure 5, and it includes:

(1) Thresholding of gait cyclograms—Only observations (points in time) where principal components contribute significantly have been extracted and analyzed; namely, the threshold values for the squared cosine of the angle *θ* which was set heuristically to 0.8 ($cos_{PC_{1}}^{2}>0.8$ and $cos_{PC_{2}}^{2}>0.8$).

(2) Estimation of the distribution density applying nonparametric kernel density estimation (KDE) [51] on the angle *θ* (obtained after thresholding in the last stride)—KDE was obtained for nine groups of data separately: H_2_, H_1.6_, H_1_, H_0.8_, H_0.4_ (both legs analyzed together for healthy subjects with the following walking speeds: $2\;\frac{m}{s}$, $1.6\;\frac{m}{s}$, $1\;\frac{m}{s}$, $0.8\;\frac{m}{s}$, $0.4\;\frac{m}{s}$, respectively), P_p.b_ and P_p.a_ (patients’ paretic legs before and after therapy), P_np.b_ and P_np.a_ (patients’ nonparetic legs before and after therapy). Shapiro–Wilk normality test [52] was used to check the (non)normality of the distributions.

(3) Clustering of distribution density to three clusters *θ*_1_, *θ*_2_, and *θ*_3_ (related to three temporal components) for each of nine groups—distribution density was smoothed by the bandwidth parameter. The bandwidth of the kernel is a free parameter that exhibits a strong influence on the resulting estimate; it is the real positive number that defines the smoothness of the density plot. The formula used to calculate optimal bandwidth parameter *bw* for each group is shown in Equation (2) [53].

(2) $$bw=\frac{0.9*\min\left(\sqrt{Var\left(X\right)},\;\frac{IQR\left(X\right)}{1.349}\right)}{\sqrt[{5}]{n}},$$

where *n* is the number of observations of *X*, *Var(X)* is its variance, and *IQR(X)* is the interquartile range. Cluster limits were extracted as local minimums of the bandwidth smoothed distribution density.

(4) Statistical analysis—Mann–Whitney U nonparametric test was performed to determine whether the same clusters (detected temporal synergies) differ statistically between patients and healthy groups [54]. Wilcoxon test for partially matched two sample data (the combination of Wilcoxon signed-rank statistics for paired data and Mann–Whitney U statistics) was used to compare healthy groups for different speeds [55]. The same test was used to compare patients before and after therapy. Finally, it was analyzed whether the statistically significant results before and after therapy can be assessed based on temporal synergism and compared the effects to conventional parameters (Section 2.6). The significance level was *p* = 0.001 for estimating the statistically significant differences.

### 2.6. Conventional Gait Analysis

The threshold method extracted the swing and stance phases for each gait session. The threshold was set to be 5% of the sum of all GRF signals in each insole divided by the number of force sensors, which was 5. The signals were filtered by a low-pass Butterworth filter, third order, with a cut-off frequency of 5 Hz. For each stride, stance and swing durations were calculated as a percentage of the gait cycle. In addition, since gait after stroke is characterized by high asymmetry, four symmetry measures were calculated for both the swing and stance phase, as in Equations (3)–(6). These measures were used to assess therapy impact on stroke patients [56,57].

(3) $$\mathrm{Symmetry}\;\mathrm{ratio}\;(\mathrm{SR}):\;\frac{T_{left}}{T_{right}},$$

(4) $$\mathrm{Symmetry}\;\mathrm{index}\;(\mathrm{SI}):\;\left(\frac{\left|T_{left}-T_{right}\right|}{0.5*\left(T_{left}+T_{right}\right)}\right)*100\%,$$

(5) $$\mathrm{Gait}\;\mathrm{asymmetry}\;(\mathrm{GA}):\;\ln(\frac{T_{left}}{T_{right}})\;*100\%,$$

(6) $$\mathrm{Symmetry}\;\mathrm{angle}\;(\mathrm{SA}):\;\frac{45^{{}^{\circ}}-\arctan\left(\frac{T_{left}}{T_{right}}\right)}{90^{{}^{\circ}}}*100\%,$$

where $T_{left}$ is the duration of the specific gait phase (stance or swing) for the left leg, and $T_{right}$ is the duration of the specific gait phase (stance or swing) for the right leg.

Whether statistically significant results could be assessed before vs. after therapy was assessed using the Wilcoxon test for partially matched two-sample data. The significance level was *p* = 0.001 for estimating the statistically significant differences.

## 3. Results

### 3.1. PCA Cyclograms

**Figure 6:**
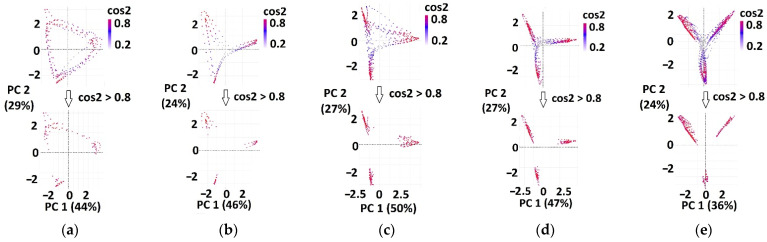
Examples of overlapped cyclograms for one healthy subject’s gait session (**top**) and thresholded cyclograms (**bottom**). Extracted (red) observations on thresholded cyclograms correspond to temporal synergies in PCA space, for the following gait speeds: (**a**) $2\;\frac{m}{s}$; (**b**)$\;1.6\;\frac{m}{s}$; (**c**) $1\;\frac{m}{s}$; (**d**)$\;0.8\;\frac{m}{s}$; (**e**)$\;0.4\;\frac{m}{s}$. In brackets, the percentages of explained variance of the specific principal component (*PC*_1_ or *PC*_2_) are shown.

Figure 6 (top) presents an example of overlapped cyclograms in a healthy subject for gait sessions with different gait speeds. For healthy subjects, the signals from sensors mounted on left and right feet were analyzed together. Figure 6 (bottom) shows thresholded cyclograms ($cos_{PC_{1}}^{2}>0.8$ and $cos_{PC_{2}}^{2}>0.8$, as explained in Section 2.5) that contain observations where temporal synergies are activated.

**Figure 7:**
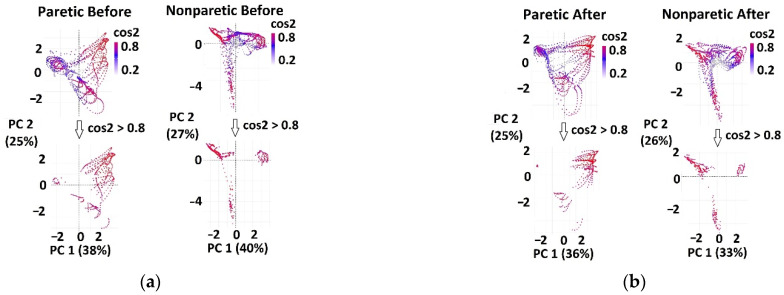
Examples of overlapped cyclograms for one patient (top) and thresholded cyclograms (bottom). Extracted (red) observations on thresholded cyclograms correspond to temporal synergies in PCA space: (**a**) before therapy (the paretic leg is in the left column, the nonparetic leg is in right column), and (**b**) after therapy (the paretic leg is in the left column and the nonparetic leg is in right column). In brackets, the percentages of explained variance of the specific principal component (*PC*_1_ or *PC*_2_) are shown.

Cyclograms for patients’ paretic and nonparetic sides, before and after therapy, were separately analyzed (Figure 7).

Angles *θ* were calculated by Equation (1) for each observation on thresholded cyclograms (for red points in Figure 6 bottom and Figure 7 bottom). Arrays of angles’ values for each of nine groups (H_2_, H_1.6_, H_1_, H_0.8_, H_0.4_, P_p.b_, P_np.b_, P_p.a_, P_np.a_) were further used as an input for KDE.

### 3.2. Temporal Synergies Extracted by KDE

**Figure 8:**
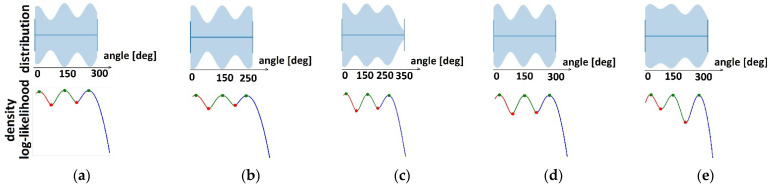
Clustering of temporal components in healthy subjects for the following gait speeds (red dots are cluster limits): (**a**) $2\;\frac{m}{s}$; (**b**)$\;1.6\;\frac{m}{s}$; (**c**) $1\;\frac{m}{s}$; (**d**)$\;0.8\;\frac{m}{s}$; (**e**)$\;0.4\;\frac{m}{s}$. Top graphics represent distribution densities, and bottom graphics represent bandwidth-smoothed distribution densities.

**Figure 9:**
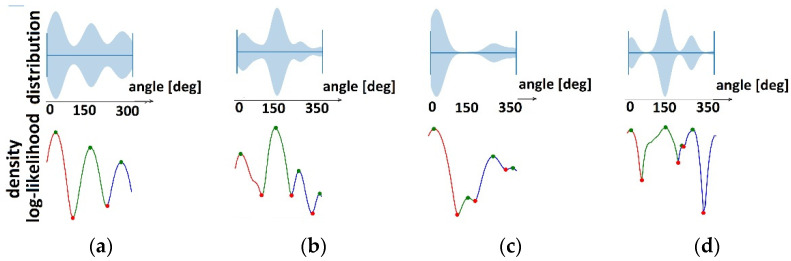
Clustering of temporal components (red dots are cluster limits) in patients for: (**a**) Paretic legs before therapy; (**b**) Nonparetic legs before therapy; (**c**) Paretic legs after therapy; (**d**) Nonparetic legs after therapy. Top graphics represent distribution densities, and bottom graphics represent bandwidth-smoothed distribution densities.

KDE was used to detect temporal synergies (clusters in time) for each of the nine groups. The cluster limits were estimated (Table 3) as local minimums in bandwidth-smoothed density distribution plots (red dots in Figure 8 and Figure 9).

In Table 3, cluster limits in distribution density of angle values *θ*_1_, *θ*_2_, and *θ*_3_ are shown in degrees for healthy subjects and patients before and after therapy.

Based on Equation (1), the mean values and standard deviations of the *θ*_1_, *θ*_2_, and *θ*_3_ angles in cyclograms (i.e., the significant contribution of activation of the first two principal components) are shown in Table 3 for all patients and healthy subjects with different speeds. It could be noticed that the angles were shifted in time by approximately one-third of the walking cycle. These angles quantify temporal activations of gait synergies.

### 3.3. Comparison of Synergies between Different Speeds in Healthy Subjects

No significant differences were found between H_2_, H_1.6_, H_1_, H_0.8_, H_0.4_ groups for *θ*_1_, *θ*_2_, and *θ*_3_ (*p* > 0.001).

### 3.4. Comparison of Synergies between Patients and Healthy Subjects

Table 4 shows the results of statistic tests between healthy groups (H_2_, H_1.6_, H_1_, H_0.8_, H_0.4_) and patients (P_p.b_, P_np.b_, P_p.a_, and P_np.a_). Significant differences were found in all angles (*θ*_1_, *θ*_2_, and *θ*_3_) between all healthy groups and paretic leg gait before therapy (P_p.b_). After therapy, the shift in the angle towards a healthy angle can be found in some angles, specifically for the lowest gait speed ($0.4\;\frac{m}{s}$), which is the most similar to the speed of the patient’s gait after stroke [58]. 

### 3.5. Comparison of Synergies between Patients before and after Therapy

**Figure 10:**
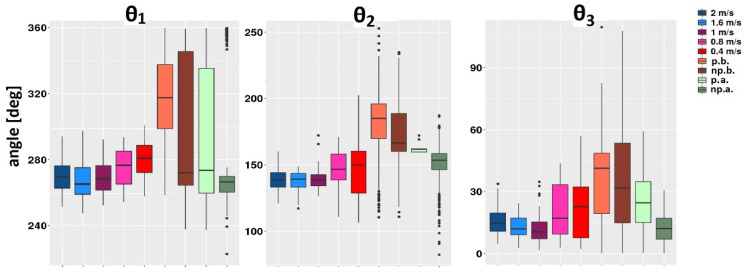
Temporal synergies (angles *θ*_1_, *θ*_2_, and *θ*_3_) for all groups.

The significant differences in patients before and after therapy were found in all angles *θ*_1_, *θ*_2_, and *θ*_3_ (*p* < 0.001). The boxplots for each angle *θ*_1_, *θ*_2_, and *θ*_3_ for all nine groups are shown in Figure 10. The temporal synergies (angles) shift can be observed after therapy towards healthy synergies.

**Figure 11:**
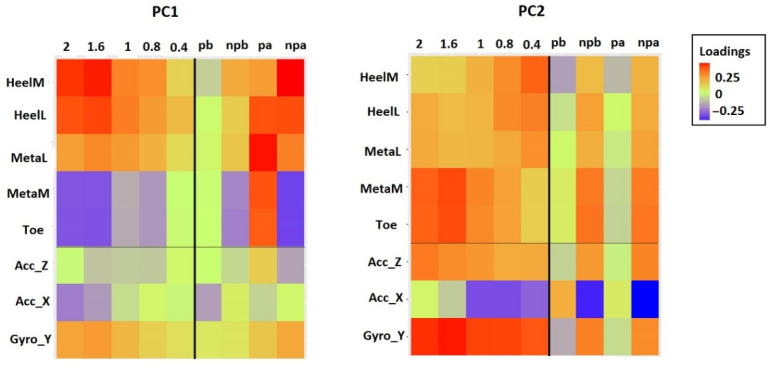
Weighting coefficients of the *PC*_1_ and *PC*_2_ for all groups. Coefficients are plotted on a color-coded scale.

Additionally, it is important to consider the relative weight of each dynamic’s signal, which is called loading. Loadings from the first two principal components are interpreted as the coefficients of the linear combination of the input variables from which the principal components are constructed. The relative strength of the effect of each factor on an input signal is given by this weighting coefficient. For each input signal, the mean weighting coefficients (loadings) of the first two components were obtained by averaging the values across all subjects for specific gait speed [19]. Figure 11 shows average weighting coefficients for all nine groups. Some input variables loaded highly on the specific component, such as angular velocity Gyro_Y on *PC*_2_. Most of the input variables are loaded on both components. The noticeable gradual change of weightings with gait speed can be noticed in loadings on both *PC*_1_ and *PC*_2_. For patients, it could be noticed that loadings in *PC*_1_ are approaching values from healthy subjects’ gaits.

### 3.6. Comparison with Conventional Methods

**Figure 12:**
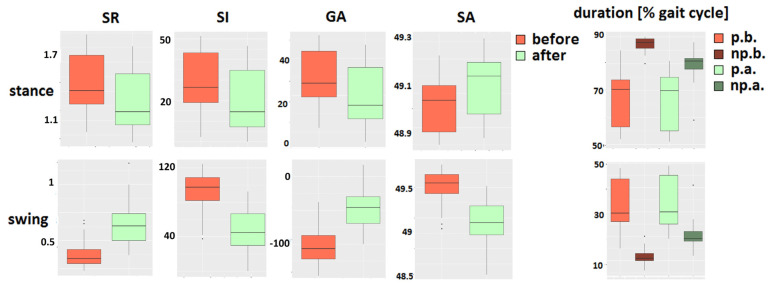
Boxplots of symmetry and temporal parameters for all patients’ groups.

In Figure 12, boxplots for symmetry (SR, SI, GA, SA) and temporal (duration) parameters of stance and swing gait phases are shown.

No significant differences between gait before and after therapy with the paretic leg could be found in swing or stance duration (*p* = 0.94). A significant difference has been found in swing and stance durations in nonparetic leg gait. Furthermore, this significant difference was reflected in swing symmetry parameters since it is proportional to the ratio of paretic and nonparetic leg parameters (*p* < 0.001) but not in the stance symmetry parameters (*p* = 0.017–0.019).

Unlike temporal parameters for the paretic leg, by observing the temporal synergies parameters (Equation (1)), the statistically significant differences for the paretic leg after therapy were found compared to before therapy (*p* < 0.001, Figure 10).

## 4. Discussion

This paper proposes a new method for detecting temporal gait synergies in dynamic space using PCA without recording muscle activity. The foot trajectory has been represented with respect to time in the PCA cyclogram space. The foot dynamics reflect the muscle activity but in a more straightforward way. Analyzing the dynamics of the endpoint—i.e., foot—is important since it is assumed that the control of limb dynamics, instead of muscle activity, would help ensure whole-body mechanical stability and energy [59]. The control of limb segment motion may happen by encoding the limb endpoint dynamics.

The inputs for PCA were GRF signals measured at the lateral and medial heel, lateral and medial metatarsal, and toe on each foot, as well as the accelerations and angular velocities measured at the rear part of each foot. The Gait Teacher system is easy to use, wearable, and relatively cheap compared to EMG-based and other gait analysis systems. It can be used in everyday life, not just in a hospital environment [60,61]. The clinician does not need the training to manage the system, unlike EMG equipment, where electrode montage and data acquisition are more time-consuming. The system’s set-up comes down to putting on shoes with insoles and following the simple interface. Due to the high level of impairment of patients in the subacute phase of stroke [62], it is crucial to provide fast and straightforward screening to make the gait evaluation more comfortable and effortless for patients.

PCA used whole preprocessed gait session recordings as an input. There was no gait segmentation before or after PCA; therefore, there was no loss of information due to the interpolation to the specific time base or incorrect stride segmentation. The problem of gait phase detection accuracy and reliability was bypassed by observing the whole gait session and evaluating the sequence at once. Unlike when calculating symmetry and temporal parameters, valuable data were lost due to incorrect gait event detection.

In this paper, three temporal activations of synergies were extracted (three modules, related to angles *θ*_1_, *θ*_2_, and *θ*_3_). Statistical tests proved the differences between healthy and patient gait before therapy and confirmed that the temporal synergies are invariant in healthy gait, regardless of different gait speeds, Table 4. These temporal activations are shifted 30% in time, which agrees with previous studies. Researchers have also claimed that three main temporal components from a set of five are also shifted by approximately 30% in time. Additionally, the existence of three synergies was statistically confirmed. The time shift of synergies was not significantly different in healthy gait for different speeds, which was not aligned with the observation from the literature [19].

The rehabilitation helps recovery of cortical neuronal networks controlling gait, and the re-emergence of healthy synergies can be noticed [63]. In this paper, synergies have been analyzed before and after therapy for stroke patients, and synergies were also compared to healthy subjects. Before the therapy, there were significant differences in all three temporal synergies compared to healthy gait. This can be explained by a change in double limb support and single limb support duration [64]. After therapy, the temporal activations ‘moved’ closer (i.e., cyclogram has rotated towards healthy cyclogram orientation) to the activations of lower speed healthy gait. The time-shift of specific synergies towards healthy values was statistically confirmed for synergies because of the FES therapy, Table 4. To the best of our knowledge, no prior studies have statistically compared temporal synergies from dynamics perspective between stroke hemiparetic gait before and after therapy with different healthy gait speeds.

On the other side, an observation can be reported about the second module related to the angle *θ*_2_ in the paretic leg before and after therapy, indicating less complex locomotor control of the affected side (Figure 7). The reduction in observations for the second module could be noticed because the same amount of variance can be explained with fewer synergies. These results agree with previous findings of the relation between less complex control and poorer walking performance [17]. A decreased number of synergies (‘disappearing’ of the second module, from three to two synergies) in the paretic leg can be explained by the merging of synergies [17,65,66]. This decrease is explained by the greater cohesion between the parts of the body and the generally reduced complexity of movement due to injury. The merging of synergies was shown in paretic gait after the therapy, indicating the possible development of abnormal synergies [17,67].

The difference in the distribution of weightings reflects complex motor coordination, although the temporal synergies are consistent (Figure 11). This means that even though the input variables had a different contribution to each PC, the activations of PCs in time remained the same. It could be noticed that some weightings in the paretic leg after therapy became more exaggerated than in a healthy one, such as lateral metatarsal force weighting on *PC*_1_ (Figure 11). This aligns with the possible development of abnormal synergies (merging of synergies) but needs further investigation.

By applying PCA, it was possible to characterize better the specific features of gait disorders in relation to commonly used techniques [40]. Therefore, the conventional temporal and symmetry parameters were also calculated. The proposed method was more advantageous than conventional gait analysis since the statistical test proved the significant difference in the paretic leg, which was not observed in temporal or symmetry parameters (Figure 12). After the therapy, symmetric gait may not be the only measure of therapy success and may not reveal the complete picture [68]. On the other hand, maximal gait variability was preserved by using PCA, and variability is a complementary way of quantifying locomotion and monitoring rehabilitation effects [69,70,71].

Other studies have examined the possibilities for follow-up of stroke patients based on the analysis of temporal muscle synergies [72]. Abnormal patterns of muscle synergies were used to provide additional measures for clinicians during various therapy sessions, such as robotic-assisted, conventional gait, or FES-cycling training [73,74]. Whether temporal synergies indicate the gait recovery of stroke patients is still arguable [65,73,74,75]. The present study showed significant changes in the temporal synergies during the rehabilitation from a dynamics perspective without considering muscle synergies.

The detection of temporal synergies from a dynamic perspective is helpful for gait assessment. Visually monitoring 2D cyclograms is a robust and straightforward qualitative measure for clinicians. The values of the proposed *θ* angles—i.e., temporal synergies—are quantitative measures of gait performance. The rotation of a 2D cyclogram (the change in temporal synergy) is a direct and simple measure that clinicians can use to assess gait performance by comparing values with healthy temporal synergies. As a result, clinicians will better understand and follow up with the therapy’s effect on gait after a stroke. Whether the gait synergies represent an input or an output of neuromuscular control is still a point of debate [76,77]. Nevertheless, defining changes in gait from a dynamic systems perspective can be useful in rehabilitation for clinical gait assessments [41,78].

The performed study has several limitations. First, the COVID-19 pandemic restrictions caused a lack of participants following the rehabilitation protocol, and for that reason, a limited number of stroke patients were included in the study. Future studies will include a larger patient population. Additionally, for the therapy efficiency assessment, data could be acquired before and after different therapy protocols [73]. Second, the study was underpowered when comparing healthy subjects and patients since they were unmatched by confounding factors, such as age and gender. Future studies will also include matched healthy and patient groups by confounding factors. Third, the wearable device used in the study has lower accuracy and reliability than the gold standard optoelectronic systems with force platforms. However, the trade-off between good performance characteristics and high cost should also be considered [79]. Finally, the question may be asked whether data loss due to PCA affects the results. Even though the initial dataset of 11 signals per leg contains more information than two PCs, the valuable information about the gait variability is kept using PCs. This dimensionality reduction imitates the problem of neural control, where many input signals fire one control signal [41]. More synergies could be observed, and more data information could be kept by adding additional components and creating three-dimensional cyclograms. Nonetheless, observing cyclograms in 2D coordinate frames (and monitoring only *θ* angles) is more convenient for the clinician than monitoring the higher dimensionality graphs and parameters. The presented concept of 2D PCA-based temporal synergies assessment is suitable for near real-time monitoring purposes and can be used to improve the current clinical tools for gait assessment in the future.

## 5. Conclusions

In this paper, an innovative method for directly observing the limb’s endpoint dynamics and detecting temporal synergies during walking with different speeds is proposed, without stride extraction, and without using EMG recordings. Furthermore, the hypothesis about invariant temporal dynamic synergies was statistically confirmed, and the potential use of this information in practical gait assessment during rehabilitation after stroke was highlighted.

## Figures and Tables

**Table 1:** Subject characteristics.

Variable	Mean ± SD	
	Healthy Subjects (*n* = 14)	Patients (*n* = 5)
Age (years)	34.8 ± 12.6	61 ± 5.1
Gender	8 male, 6 female	1 male, 4 female
Total body mass (kg)	73.3 ± 12.7	78.4 ± 9.2
Height (m)	1.78 ± 0.08	1.7 ± 0.05
BMI (kg/m^2^)	23 ± 2.18	28.18 ± 3.6
Affected side	-	4 left, 1 right

**Table 2:** Cadence for various speeds and stride lengths on a path of 10 m (SPM = strides per minute).

SPM	$2\;\frac{m}{s}$	$1.6\;\frac{m}{s}$	$1\;\frac{m}{s}$	$0.8\;\frac{m}{s}$	$0.4\;\frac{m}{s}$
0.5 m	240	192	120	96	48
0.75 m	160	128	80	64	32
1 m	120	96	60	48	24

**Table 3:** Cluster limits (temporal components) in distribution density of angle values *θ*_1_, *θ*_2_, and *θ*_3_, mean ± standard deviation, all expressed in degrees.

		Healthy Subjects					Patients before Therapy		Patients after Therapy	
		H_2_	H_1.6_	H_1_	H_0.8_	H_0.4_	P_p.b_	P_np.b_	P_p.a_	P_np.a_
$\theta_{1}$	cluster limits	[198–360]	[198–360]	[198–360]	[206–360]	[213–360]	[257–360]	[235–360]	[191–360]	[206–360]
	mean ± SD	265 ± 14.9	262 ± 15.7	266 ± 7.9	271 ± 15.8	276 ± 12.8	318 ± 24.4	295 ± 38.6	291 ± 40.3	272 ± 27.6
$\theta_{2}$	cluster limits	[73–197]	[73–197]	[73–197]	[88–205]	[81–212]	[110–256]	[110–234]	[118–190]	[59–205]
	mean ± SD	138 ± 8.9	138 ± 7.5	140 ± 9.7	147 ± 14	147 ± 22.6	183 ± 26.1	172 ± 18.4	162 ± 4.3	152 ± 11.7
$\theta_{3}$	cluster limits	[0–72]	[0–72]	[0–72]	[0–87]	[0–80]	[0–109]	[0–109]	[0–117]	[0–58]
	mean ± SD	16 ± 7	13 ± 5.2	13 ± 8.3	21 ± 12.9	23 ± 16.7	36 ± 18.9	39 ± 12.7	25 ± 12.7	12 ± 6.9

**Table 4:** Statistical differences between healthy and patient groups (before and after therapy).

	Patients		P_p.b._	P_np.b._	P_p.a._	P_np.a._
Healthy						
$H_{2}$		$\theta_{1}$	0 *	0 *	0 *	0 *
		$\theta_{2}$	0 *	0 *	0 *	0 *
		$\theta_{3}$	0 *	0 *	0 *	0.008
$H_{1.6}$		$\theta_{1}$	0 *	0 *	0 *	0 *
		$\theta_{2}$	0 *	0 *	0 *	0 *
		$\theta_{3}$	0 *	0 *	0 *	0.332
$H_{1}$		$\theta_{1}$	0 *	0 *	0.001	0.115
		$\theta_{2}$	0 *	0 *	0 *	0 *
		$\theta_{3}$	0 *	0 *	0 *	0.594
$H_{0.8}$		$\theta_{1}$	0 *	0 *	0.022	0.045
		$\theta_{2}$	0 *	0 *	0 *	0.004
		$\theta_{3}$	0 *	0 *	0.024	0.002
$H_{0.4}$		$\theta_{1}$	0 *	0.588	0.301	0.001
		$\theta_{2}$	0 *	0 *	0.008	0.138
		$\theta_{3}$	0 *	0.002	0.190	0.001

* *p* < 0.001.

## Data Availability

The data presented in this study are available on request from the corresponding author.
